# Body image and sport: a qualitative study

**DOI:** 10.1186/2050-2974-1-S1-O51

**Published:** 2013-11-14

**Authors:** Joanna McCormack, Anita Star, Jacqueline Beadle, Nadia Maartens

**Affiliations:** 1 Griffith University, Australia

## 

Previous research has overwhelmingly established a relationship between sport participation and the development of positive body image (BI) (Richman & Schaffer (2000), Shaffer & Wittes (2006) and Greenleaf et al (2009). However a number of studies have indicated some women are particularly vulnerable to the development of poor BI in relation to sports participation (Slater & Tiggeman, 2011). Due to the conflicting evidence, we have used an interpretive phenomenological framework to qualitatively explore women's experiences. Specifically we aimed to understand the perceived impact of sports participation and related commentary during childhood on adult body image. Participants were given the opportunity to suggest interventions which would improve their experiences of sports in relation to BI.

Women indicated they started to think about their bodies from as young as 6 and 7. They remembered critical incidences that occurred during childhood sports which they perceived to influence their BI either in a positive or negative way. The awareness of their bodies has often come about through commentary from family or coaches, or through sporting uniforms. Suggestions for interventions have included mandatory education on overall health and food from as young as 6 in primary schools.

This abstract was presented in the **Body Image** stream of the 2013 ANZAED Conference.

